# Intravenous Iron Repletion Does Not Significantly Decrease Platelet Counts in CKD Patients with Iron Deficiency Anemia

**DOI:** 10.1155/2013/878041

**Published:** 2013-02-12

**Authors:** Neville R. Dossabhoy, Rebecca Gascoyne, Steven Turley

**Affiliations:** ^1^Department of Medicine, Louisiana State University Health Sciences Center, Shreveport, LA 71130-3932, USA; ^2^Department of Medicine, Overton Brooks Veterans Affairs Medical Center, Shreveport, LA 71101, USA

## Abstract

*Purpose*. We sought to investigate the effect of IV iron repletion on platelet (PLT) counts in CKD patients with iron deficiency anemia (IDA). *Methods*. We conducted a retrospective chart review, including all patients with CKD and IDA who were treated with iron dextran total dose infusion (TDI) between 2002 and 2007. Patient demographics were noted, and laboratory values for creatinine, hemoglobin (Hgb), iron stores and PLT were recorded pre- and post-dose. *Results*. 153 patients received a total of 251 doses of TDI (mean ± SD = 971 ± 175 mg); age 69 ± 12 years and Creatinine 3.3 ± 1.9 mg/dL. All CKD stages were represented (stage 4 commonest). Hgb and Fe stores improved post-TDI (*P* ≪ 0.001). There was a very mild decrease in PLT (pre-TDI 255 versus post-TDI 244, *P* = 0.30). The mild reduction in PLT after TDI remained non-significant (*P* > 0.05) when data was stratified by molecular weight (MW) of iron dextran used (low versus high), as well as by dose administered (<1000 versus ≥1000 mg). Linear regression analysis between pre-dose PLT and Tsat and Fe showed R2 of 0.01 and 0.04, respectively. *Conclusion*. Correction of iron deficiency did not significantly lower PLT in CKD patients, regardless of MW or dose used. Correlation of PLT to severity of iron deficiency was very weak.

## 1. Introduction 

Intravenous (IV) iron has become a cornerstone of therapy for anemia in patients with chronic kidney disease (CKD)—both before hemodialysis and for those on hemodialysis. One of the earliest intravenous (IV) compounds used to treat iron deficiency anemia was iron dextran; however, its popularity as a first-line agent had waned considerably secondary to concerns regarding its safety and the introduction of newer compounds on the market.

Iron deficiency often leads to reactive thrombocytosis [1–5], with platelet counts sometimes as high as 500,000 to 700,000 cells/mm^3^. The etiology of the thrombocytosis in iron deficiency remains uncertain. Platelet (PLT) counts have been reported to vary directly with the severity of iron deficiency in chronic hemodialysis patients. This has led some to hypothesize that iron deficiency may produce a relative thrombocytosis which may contribute to the thrombotic events noted in clinical trials of erythropoiesis stimulating agents (ESA's) in CKD patients.

Even more interestingly, PLTs were reduced after IV iron administration in hemodialysis patients in the recent DRIVE study [6–8]. However, the PLT count remained unchanged in patients in the treatment arm not given IV Iron. In a recent NKF abstract, Besarab et al. reported that correction of iron deficiency lowers PLT in nondialysis CKD patients [9]. 

In this study, we sought to confirm the reduction in PLT by IV iron repletion (using iron dextran total dose infusion) in CKD patients with iron deficiency anemia. 

## 2. Patients and Methods

The study was conducted at the Overton Brooks VA Medical Center in Shreveport, Louisiana. Approval was obtained from our institutional review board and research and development committees. We conducted a retrospective chart review spanning a 5 year-period (2002–2007), including all patients with chronic kidney disease and iron-deficiency anemia who were treated with iron dextran total dose infusion (TDI). It should be noted that, at our medical center, iron dextran was one of the first-line IV iron agents on the formulary in the years under study. Iron dextran TDI diluted in normal saline was administered intravenously (IV) over 4–6 hours (if there was no reaction to an initial test dose of 25 mg). Patient demographics and comorbid conditions were noted, and laboratory values for creatinine, platelet count (PLT), hemoglobin (Hgb), hematocrit (Hct), serum iron (Fe), % transferrin saturation (Tsat), and ferritin (Ftn) were recorded before dose and after dose. The after dose values were measured 10–120 days post-infusion, when the patient came back to the clinic or hospital for follow-up care.

Data were analyzed using Student's *t*-test to compare mean values. Linear regression analysis was performed to elucidate possible relationships between pre-dose PLT and Tsat and pre-dose PLT and serum Fe. Microsoft Excel version 2007 was used to perform the above statistical testing (Microsoft Corporation, Redmond, Washington, USA).

## 3. Results

153 patients received a total of 251 doses of TDI (mean ± SD = 971 ± 175 mg). The patients' age was 69 ± 12 years and creatinine at enrollment was 3.3 ± 1.9 mg/dL. All stages of CKD were represented, though stage 4 was the commonest-see Table 1. Hemoglobin and Fe stores improved post-TDI (*P* < 0.001); see Table 2. 

There was a very mild decrease in PLT (pre-TDI 255 ± 114 versus post-TDI 244 ± 108, *P* = 0.30) for the group as a whole. The different stages of CKD were analyzed separately to test if there was a significant difference between preinfusion and postinfusion PLT counts. We did not find a statistically significant difference in any CKD stage. 

The mild reduction in PLT after TDI remained nonsignificant (*P* > 0.05) when data was stratified by molecular weight (MW) of iron dextran used (low versus high), as well as by dose administered (<1000 versus ≥1000 mg). Patients who were administered the low MW dextran (*N* = 140) had a mean pre-TDI PLT of 248 ± 108 and mean post-TDI count of 231 ± 91, with *P* = 0.19. For patients receiving the high MW iron dextran (*N* = 110), the mean pre-TDI PLT was 261 ± 121 and the mean post-TDI 258 ± 125, with a *P* value of 0.87.

Patients were further categorized by the dose of iron dextran administered (<1000 versus ≥1000 mg). For doses <1000 mg (*N* = 29), the pre-TDI PLT count was 254 ± 143 and the post-TDI value 258 ± 178 (*P* = 0.93). For doses ≥1000 mg (*N* = 221), the pre-TDI PLT count was 255 ± 110 and the post-TDI count was 242 ± 97, with a *P*-value of 0.22. 

**Figure 1:**
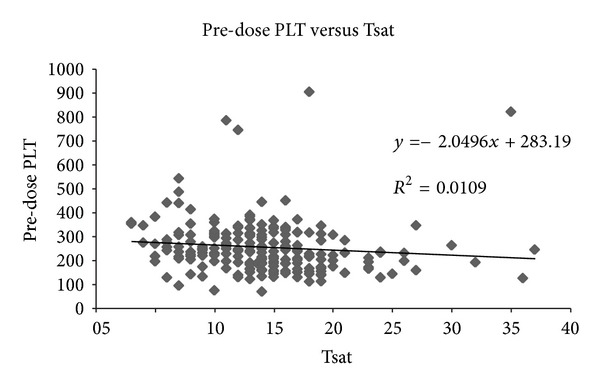
Linear regression analysis of pre-dose PLT versus Tsat (*R* ^2^ = 0.01).

**Figure 2:**
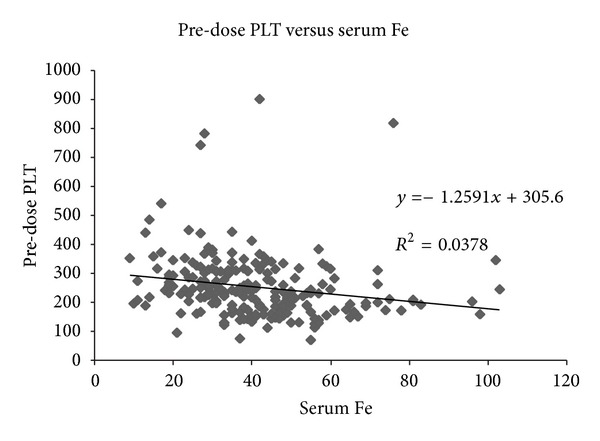
Linear regression analysis of pre-dose PLT versus serum Fe (*R* ^2^ = 0.04).

Linear regression analysis was performed, which showed *R* ^2^ of 0.01 between pre-dose PLT and Tsat, whilst that between pre-dose PLT and serum Fe revealed *R* ^2^ of 0.04 (Figures 1 and 2). The slope was negative, namely, that the lower the iron stores, the higher the PLT count, but the correlation was very weak.

We further looked at the percentage of patients who had PLT counts of 350 or higher, since these are the patients who would be expected to have a higher likelihood of thrombotic events. 11.6% of the patients had a baseline (pre-TDI) PLT of 350 or higher, whereas 10.6% satisfied this criterion post-TDI (*P*-value < 0.0001 by chi-square test as well as by Fischer's exact test). 

We then divided patients into two groups, based on whether their preinfusion PLT count was 350 or higher, or less than 350. The number of such patients with PLT ≥350 was small, 25 patients. There was no statistically significant difference between preinfusion and postinfusion PLT counts in this group. We also did a similar analysis in patients whose PLT count was less than 350; here too, no statistically significant difference between preinfusion and postinfusion PLT counts was found.

## 4. Discussion

Iron deficiency may lead to reactive thrombocytosis [1]. Unexplained thrombocytosis has been described, sometimes with platelet counts in the range of 500,000 to 700,000 cells/mm^3^. The etiology of the thrombocytosis in iron deficiency remains a mystery. Megakaryocytes and normoblasts are derived from a common committed progenitor cell, the CFU-GEMM (colony-forming unit, granulocytic, erythroid, myelomonocytic). Thrombopoietin, the molecule that stimulates the growth of megakaryocytes and the production of platelets, is structurally similar to erythropoietin, the molecule that promotes red cell development. Some investigators have speculated that the elevated levels of erythropoietin in patients with iron deficiency anemia might modestly increase platelet production by cross-reacting with the thrombopoietin receptor. On the other hand, investigations with recombinant human erythropoietin and thrombopoietin indicate that cross-reactivity does not occur amongst these recombinant products (analogues).

The thrombocytosis in iron deficiency is usually mild, although severe iron deficiency has been documented as causing marked thrombocytosis (>1 million platelets/mm^3^) complicated by central retinal vein occlusion [2]. Two cases of cerebral venous sinus thrombosis associated with iron deficiency and a normal platelet count have also been reported [3]. Among six children with iron deficiency and ischemic stroke or venous thrombosis, four had concomitant thrombocytosis [4]. According to a recent review by Stolz et al., severe anemia, thrombocytosis, and hypercholesterolemia are independent risk factors for cerebral venous thrombosis [5]. 

Platelet counts (PLTs) have been reported to vary directly with the severity of iron deficiency in chronic hemodialysis (HD) patients. Even more interestingly, PLTs were reduced after IV iron administration in the recent DRIVE study [6–8]. In a recent NKF abstract, Besarab et al. had reported that correction of iron deficiency lowers PLTs [9]. This has led some to hypothesize that iron deficiency may produce a relative thrombocytosis that may contribute to thrombotic events noted in clinical trials of ESA's in CKD patients. According to this view, iron deficiency is a risk factor for thrombocytosis that should, wherever possible, be avoided. Optimal coadministration of iron may possibly reduce the risk for ESA-driven cardiovascular events.

In our study, correlation of pre-dose PLT to indices of iron stores was very weak (*R* ^2^ = 0.01 to 0.04); and it was negative in direction (see Figures 1 and 2). Hence, the iron deficiency may produce a relative thrombocytosis in theory, but the clinical effect seems to be very insignificant. More importantly, the correction of iron deficiency did not significantly lower PLT count in our iron-deficient CKD patients regardless of MW or dose of IV iron dextran used, the stage of CKD, and the preinfusion PLT count (whether >350 or not). 

We looked at the percentage of patients who had PLT of 350 or higher, since the higher PLT count could be expected to result in a higher likelihood of thrombotic events. 11.6% of the patients had a baseline (pre-TDI) PLT 350 or higher, whereas 10.6% satisfied this criterion post-TDI (*P*-value < 0.0001). Although this percentage was “statistically significant,” the clinical significance of this is uncertain at present, given the lack of statistical significance for the change in the total PLT count and the fact that this retrospective study was not designed to follow long-term clinical cardiovascular outcomes (stroke, MI, etc.). When we divided patients into two groups, based on whether their preinfusion PLT count was ≥ 350 or less than 350, there was no statistically significant difference between preinfusion and postinfusion PLT counts in either group. 

In general, this is a negative study, but a reasonably important negative one. In contradistinction to some previous evidence and emerging popular theories, it points out the lack of significant influence on platelets counts of an effective iron supplementation (IV route). Indeed, a long line of clinical observations in the past has noted that states of anemia associated with increased endogenous erythropoietin (EPO) levels (e.g., chronic inflammation and iron deficiency) also have chronic elevation of platelets counts, unless there is a coincidental DNA synthesis impairment (megaloblastic anemia). Therefore, in a very simple sense and under select circumstances, one can view the platelet count as an “in vivo bioassay,” a surrogate of endogenous EPO levels. Anemia of CKD characterized by at least a relative deficiency of EPO was, therefore, not associated as a routine with platelet count elevations. The lack of meaningful association in this study between PLT and Tsat/serum Fe would likely imply relatively low endogenous EPO levels. In contradistinction to the above mechanism, exogenous administrations of EPO (analogues) were never shown to increase platelet counts; hence, these are very different from circumstances when endogenous EPO is stimulated. A potential confounder of this study, if any, would be an exogenous administration of EPO analogues, which could suppress endogenous secretion of EPO and may mask any platelet changes with correction of iron deficiency. A relative weakness of this study is that endogenous EPO levels were not measured; however, this is to be understood based on the retrospective nature of the study and the lack of funding. Another weakness is that the postdose values were measured 10–120 days after infusion, when the patient came back to the clinic or hospital for follow-up care. The range is somewhat wider than ideal but reflects the constraints of a retrospective study done without the inherent stringent design of a prospective trial.

The association of the recently reported PLT reduction to postulated improvement in thrombotic outcomes needs additional long-term research. Future clinical trials can be designed to answer some of the questions raised by this hypothesis generating study. Correction of iron deficiency did improve Fe stores and erythropoiesis, resulting in higher hemoglobin in the patients (Table 2). It should be noted that, at our medical center, iron dextran was one of the first-line IV iron agents on the formulary in the years under study. It is currently not a first-line agent in general practice, because of concerns regarding its safety and the introduction of newer compounds on the market. In the future, this study may be extended to study other newer and more frequently used compounds.

## 5. Conclusions

Our study confirms that IV iron dextran, given as total dose infusion, does improve Fe stores and erythropoiesis, resulting in higher hemoglobin levels.

The correlation of pre-dose platelet counts to indices of iron stores was very weak (*R* ^2^ = 0.01 to 0.04). Correction of iron deficiency did not significantly lower platelet counts in CKD patients, regardless of the molecular weight of iron dextran used or the dose administered, the stage of CKD, and the preinfusion PLT count (whether >350 or not). We also looked at the percentage of patients who had PLT 350 or higher, since these are the patients in whom one would expect to have a higher likelihood of thrombotic events. 11.6% of the patients had a baseline (pre-TDI) PLT 350 or higher, whereas 10.6% satisfied this criterion post-TDI. When we divided patients into two groups, based on whether their preinfusion PLT count was ≥ 350 or less than 350, there was no statistically significant difference between pre-infusion and postinfusion PLT counts in either group.

In general, this is a reasonably important negative study. It stands in contrast to some previous evidence and current popular theories, and it points out the lack of significant influence on platelets counts of an effective iron supplementation (IV route).

In our opinion, the association of the recently reported reduction in platelet count by IV iron repletion to a postulated improvement in thrombotic outcomes needs further and intensive long-term study. In fact, there may well be other mechanisms that better explain the increased thrombotic events noted in clinical trials of ESA' s in CKD patients in the last few years.

## Figures and Tables

**Table 1:** Distribution of IV iron dextran doses by CKD Stage.

CKD Stage	Number of doses	Percent
CKD 1	2	1%
CKD 2	2	1%
CKD 3	77	31%
CKD 4	93	37%
CKD 5	33	13%
CKD 6 (on dialysis)	44	18%

Total	251	100%

**Table 2:** Effect of iron dextran TDI on PLT, Hgb and iron stores.

	PLT	Hgb	Hct	Fe	Tsat	Ftn
Pre-TDI	255 ± 114	10.8 ± 1.4	32.5 ± 4.3	42 ± 18	15 ± 6	183 ± 228
Post-TDI	244 ± 108	11.3 ± 1.5	33.9 ± 4.7	62 ± 28	24 ± 10	436 ± 281
*P*-value	0.30	≪0.001	≪0.001	≪0.001	≪0.001	≪0.001
